# Establishment of a General NAFLD Scoring System for Rodent Models and Comparison to Human Liver Pathology

**DOI:** 10.1371/journal.pone.0115922

**Published:** 2014-12-23

**Authors:** Wen Liang, Aswin L. Menke, Ann Driessen, Ger H. Koek, Jan H. Lindeman, Reinout Stoop, Louis M. Havekes, Robert Kleemann, Anita M. van den Hoek

**Affiliations:** 1 Department of Metabolic Health Research, The Netherlands Organization for Applied Scientific Research (TNO), Leiden, The Netherlands; 2 Department of Endocrinology and Metabolic Diseases, Leiden University Medical Center, Leiden, The Netherlands; 3 TNO-Triskelion, Zeist, The Netherlands; 4 Department of Pathology, University Hospital Antwerp, University of Antwerp, Edegem, Belgium; 5 Department of Internal Medicine, Division of Gastroenterology and Hepatology, University Medical Center Maastricht, Maastricht, The Netherlands; 6 Department of Vascular Surgery, Leiden University Medical Center, Leiden, The Netherlands; Institute of Medical Research A Lanari-IDIM, University of Buenos Aires-National Council of Scientific and Technological Research (CONICET), Argentina

## Abstract

**Background and aims:**

The recently developed histological scoring system for non-alcoholic fatty liver disease (NAFLD) by the NASH Clinical Research Network (NASH-CRN) has been widely used in clinical settings, but is increasingly employed in preclinical research as well. However, it has not been systematically analyzed whether the human scoring system can directly be converted to preclinical rodent models. To analyze this, we systematically compared human NAFLD liver pathology, using human liver biopsies, with liver pathology of several NAFLD mouse models. Based upon the features pertaining to mouse NAFLD, we aimed at establishing a modified generic scoring system that is applicable to broad spectrum of rodent models.

**Methods:**

The histopathology of NAFLD was analyzed in several different mouse models of NAFLD to define generic criteria for histological assessment (preclinical scoring system). For validation of this scoring system, 36 slides of mouse livers, covering the whole spectrum of NAFLD, were blindly analyzed by ten observers. Additionally, the livers were blindly scored by one observer during two separate assessments longer than 3 months apart.

**Results:**

The criteria macrovesicular steatosis, microvesicular steatosis, hepatocellular hypertrophy, inflammation and fibrosis were generally applicable to rodent NAFLD. The inter-observer reproducibility (evaluated using the Intraclass Correlation Coefficient) between the ten observers was high for the analysis of macrovesicular steatosis and microvesicular steatosis (ICC = 0.784 and 0.776, all p<0.001, respectively) and moderate for the analysis of hypertrophy and inflammation (ICC = 0.685 and 0.650, all p<0.001, respectively). The intra-observer reproducibility between the different observations of one observer was high for the analysis of macrovesicular steatosis, microvesicular steatosis and hypertrophy (ICC = 0.871, 0.871 and 0.896, all p<0.001, respectively) and very high for the analysis of inflammation (ICC = 0.931, p<0.001).

**Conclusions:**

We established a simple NAFLD scoring system with high reproducibility that is applicable for different rodent models and for all stages of NAFLD etiology.

## Introduction

Non-alcoholic fatty liver disease (NAFLD) has become the most common chronic liver disease in developed countries, paralleling the increased prevalence of obesity during the last decades [1]–[3]. NAFLD encompasses a wide spectrum of liver pathology ranging from non-alcoholic hepatic steatosis to steatohepatitis (NASH), that can evolve into liver fibrosis, cirrhosis and its life threatening complications or hepatocellular cancer [4].

Currently, liver biopsy is still the ‘golden standard’ for NASH diagnosis in clinical setting. Liver biopsies will however often be taken in subjects at high risk, leading to a bias in the samples towards the more severe forms of NAFLD. Histological scoring systems designed for human diagnostic purposes therefore usually concentrate on facets of the later stages of NAFLD, making them less applicable for scoring early stages of NAFLD. However, in 2005, the NASH Clinical Research Network (NASH-CRN) established and validated a system of histological evaluation for the full spectrum of NAFLD that could be useful in clinical trials [5]. Importantly, this scoring system of Kleiner *et al.* was not developed for diagnosing NASH, but for monitoring histopathological changes during clinical trials. The semi-quantitative scoring system separates the grading (activity) from staging (fibrosis) [5]. The grading or NAFLD activity score (NAS) encompasses steatosis, lobular inflammation and ballooning [5], [6]. The methodology proposed for scoring the different features of NAFLD has now been widely utilized, as evidenced by its application in numerous studies. Among the studies that use the Kleiner scoring system are several preclinical animal studies. However, despite this widespread use of the scoring system in different mouse models of NAFLD, the original scoring system was intended for human samples and has never been validated for experimental rodent samples.

To this end, by comparing human liver biopsies with the liver samples of different NAFLD mouse models (HFD-fed C57BL6, MCD-diet fed C57BL6, HC-diet fed APOE*3Leiden, HFD-fed and HFC-diet fed APOE*3Leiden.CETP, HFD-fed and HFC-diet fed LDLR^−/−^.Leiden and HFD-fed KKA^y^ mice), we first assessed whether specific features of human NAFLD pathology are present in NAFLD mouse models. Additionally, and based upon the features pertaining to mouse NAFLD, we defined a set of robust histological criteria to analyze NAFLD in rodents and propose a modified Kleiner scoring system that is applicable to all stages of NAFLD etiology and that could generally be used in preclinical rodent studies because of its low inter- and intra-observer variability.

## Materials and Methods

### Human NAFLD samples

Thirty human liver biopsies were obtained during autopsy for postmortem histological analysis (Department of Pathology; Leiden University Medical Center/LUMC, Leiden, The Netherlands). Sample collection and handling was performed in accordance with the guidelines of the Committee Medical Ethics (CME, Leiden, The Netherlands) and the code of conduct of the Dutch Federation of Biomedical Scientific Societies and after permission of the institutional review board. The LUMC review board does not require ethical consent for this study as the human material in the study fulfils the criteria for “further use” i.e. the material is collected in the context of patient care and is later made available for scientific use provided that the material is used anonymously, not required for clinical use, that the patient has not objected to the further use, and that tissue handling is performed in accordance with the local and national guidelines. Cardiovascular disease was the predominant cause of death. Cases with a known or suspected alcohol abuse were excluded. Forty percent of the subjects were female and sixty percent male. The average age was 63 years. Liver samples were fixed in formalin, paraffin embedded and sections were stained with hematoxylin and eosin (H&E) and Sirius Red. Samples were scored for NAFLD (using NAS) and fibrosis stage and subsequently diagnosed as no evidence of fatty liver disease, steatosis or steatohepatitis by a clinical pathologist (A.D). In Table 1 the most important histological characteristics for the human samples are listed per diagnosed category, additional histological features are listed in S1 Table.

**Table 1:** Histological characteristics of human samples per diagnosed category.

Histological feature	Score/code	Healthy (n = 9)	NAFLD (n = 11)	NASH (n = 10)
Steatosis grade	0	9	0	0
	1	0	4	4
	2	0	6	4
	3	0	1	2
Lobular inflammation	0	9	11	0
	1	0	0	10
	2	0	0	0
	3	0	0	0
Ballooning	0	8	11	0
	1	1	0	8
	2	0	0	2
Fibrosis stage	0	6	6	2
	1	1	0	0
	1A	0	4	2
	1B	1	0	4
	1C	0	0	0
	2	1	1	1
	3	0	0	0
	4	0	0	1

Number of subjects per score/code are shown for the indicated histological features.

### Animals and induction of NAFLD

Several animal models, covering the spectrum of NAFLD from simple steatosis to NASH without or with fibrosis, were used to define robust histological criteria for experimental disease and to validate the rodent scoring system. In Table 2 the metabolic and histological characteristics of the different animal models and diet combinations are shown.

**Table 2:** Metabolic and histological characteristics of the used NAFLD mouse models.

Model	Obesity	IR	HTG	Steatosis	Hepatic inflammation	Fibrosis
C57BL6, HFD	+	+	−	+	+	−
C57BL6, MCD	−	−	−	++	++	++
E3L, HC	−	−	−	+	++	++
E3L.CETP, HFD	+	+	−	++	−	−
E3L.CETP, HFC	+	+	+	++	++	+
LDLR^−/−^.Leiden, HFD	++	++	+	++	++	++
LDLR^−/−^, Leiden, HFC	+	+	++	++	++	++
KKA^y^, HFD	++	++	+	++	−	−

Severity is indicated as + or ++, absence is indicated as −. IR: insulin resistance; HGT: hypertriglyceridemia; HFD: high fat diet; MCD: methionine and choline deficient diet; HC: high cholesterol diet; HFC: high fat and cholesterol diet.

Male C57BL/6J mice, 12 weeks of age, were obtained from Charles River and were fed a high fat diet (HFD: 45 energy% fat derived from lard, Research Diets, New Brunswick, NJ, USA) for 24 weeks (n = 10).

Male C57BL/6J mice, 4–8 weeks of age, were obtained from Charles River and were fed a methionine choline deficient diet (MCD: cat#960439; MP Biomedicals, Eindhoven, The Netherlands) for 8 weeks (n = 10).

Female APOE*3Leiden (E3L) mice [7], 17–20 weeks of age were obtained from the in-house breeding colony (TNO Metabolic Health Research, Leiden, The Netherlands) and were fed a high cholesterol diet (HC: 35 energy% fat primarily as cocoa butter, 40 energy% sucrose and 1% cholesterol, modified semi-synthetic diet based on Nishina et al., [8]) for 20 weeks (n = 10).

Human CETP transgenic mice that express cholesteryl ester transfer protein (CETP) under control of its natural flanking regions (strain 5203) [9] were obtained from Jackson laboratories (Bar Harbor, MC) and cross-bred with APOE*3Leiden mice in our local animal facility at TNO to obtain heterozygous E3L.CETP mice [10], [11]. Male E3L.CETP mice, 10–14 weeks of age, were either fed a HFD for 16 weeks (n = 10) to induce steatosis without steatohepatitis or were fed the HFD for 10 weeks, followed by 6 weeks of the HFD supplemented with 1% cholesterol (n = 10), to induce steatohepatitis.

Male LDLR^−/−^ mice [12], 13–17 weeks of age, were obtained from the in-house breeding colony (LDLR^−/−^.Leiden strain; TNO Metabolic Health Research, Leiden, The Netherlands). This strain is more sensitive to diet-induced obesity and comorbidities than regular LDLR^−/−^ mice. Mice were fed a HFD for 10 weeks, followed by 6 weeks of the HFD supplemented with 1% cholesterol (n = 10), or in a separate experiment, fed a HFD for 21 weeks (n = 10).

Male KKA^y^ mice, 5–7 weeks of age, were obtained from Jackson Laboratories and were fed a HFD for 16 weeks (n = 10).

All animals were housed in a temperature-controlled room on a 12 hour light-dark cycle and had free access to food and water. Mice were sacrificed using CO_2_ asphyxiation. Liver samples (of lobus sinister medialis hepatis and lobus dexter medialis hepatis) were collected, fixed in formalin, paraffin embedded and sections were stained with hematoxylin and eosin (H&E) and Sirius Red. Animal experiments were approved by the Ethical Committee on Animal Care and Experimentation (Zeist, The Netherlands), and were in compliance with European Community specifications regarding the use of laboratory animals.

### Defining the overlapping features of NAFLD in humans and mice

**Figure 1:**
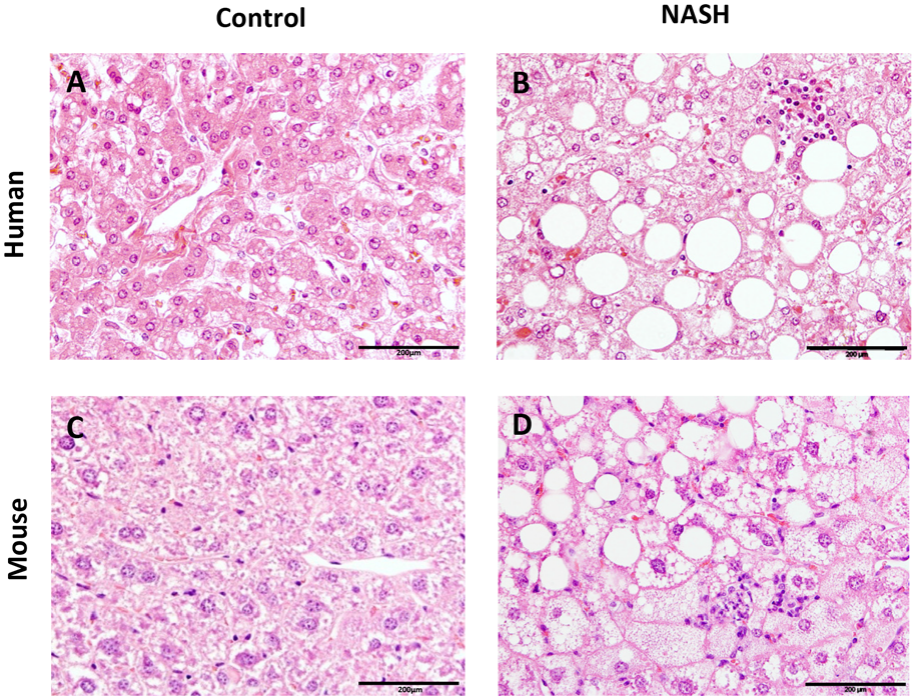
Histological photomicrographs of human NASH and NASH in mice. Liver histological cross-sections from a healthy control subject (A) and a NASH patient (B). Liver histological cross-sections from a healthy control (C) and a NASH E3L.CETP mouse (D). All photomicrographs: Hematoxylin and eosin; magnification 200x.

Using the histological scoring system of Kleiner *et al.*, tissue samples from mouse NAFLD models were compared to liver biopsy samples obtained from NAFLD patients to examine whether recognized features of human NAFLD were present in any of the experimental NAFLD models (Table 3). Liver histology photomicrographs were taken to compare histological features from human and mouse (control vs. NASH) (Fig. 1).

**Table 3:** Histological features of NAFLD in humans and presence or absence in mice.

Histological feature		Human NAFLD	Mouse NAFLD
Steatosis			
	Steatosis	**√**	**√**
	Microvesicular steatosis	**√**	**√**
Inflammation			
	Lobular inflammation (overall assessment of all inflammatory foci)	**√**	**√**
	Microgranulomas (small aggregates of macrophages)	**√**	**√**
	Large lipogranulomas	**√**	**√**
	Portal inflammation	**√**	**√**
Fibrosis		**√**	**√**
Liver cell injury			
	Ballooning cells	**√**	√*
	Acidophil bodies (hepatic apoptosis)	**√**	**-**
	Pigmented macrophages	**√**	**-**
	Megamitochondria	**√**	**-**
Other findings			
	Mallory-Denk bodies	**√**	**-**
	Nuclear glycogenation	**√**	**-**

The presence or absence of the main histological features of NAFLD as identified in humans was evaluated in several different mouse models for NAFLD (C57BL6 mice on a high fat diet or on methionine choline deficient (MCD) diet, E3L, E3L.CETP, LDLR^−/−^.Leiden, KKA^y^ mice): “**√**” presence; “−” absence of features.* Ballooning cells were occasionally observed in the models.

### Definition of histological NAFLD score

**Figure 2:**
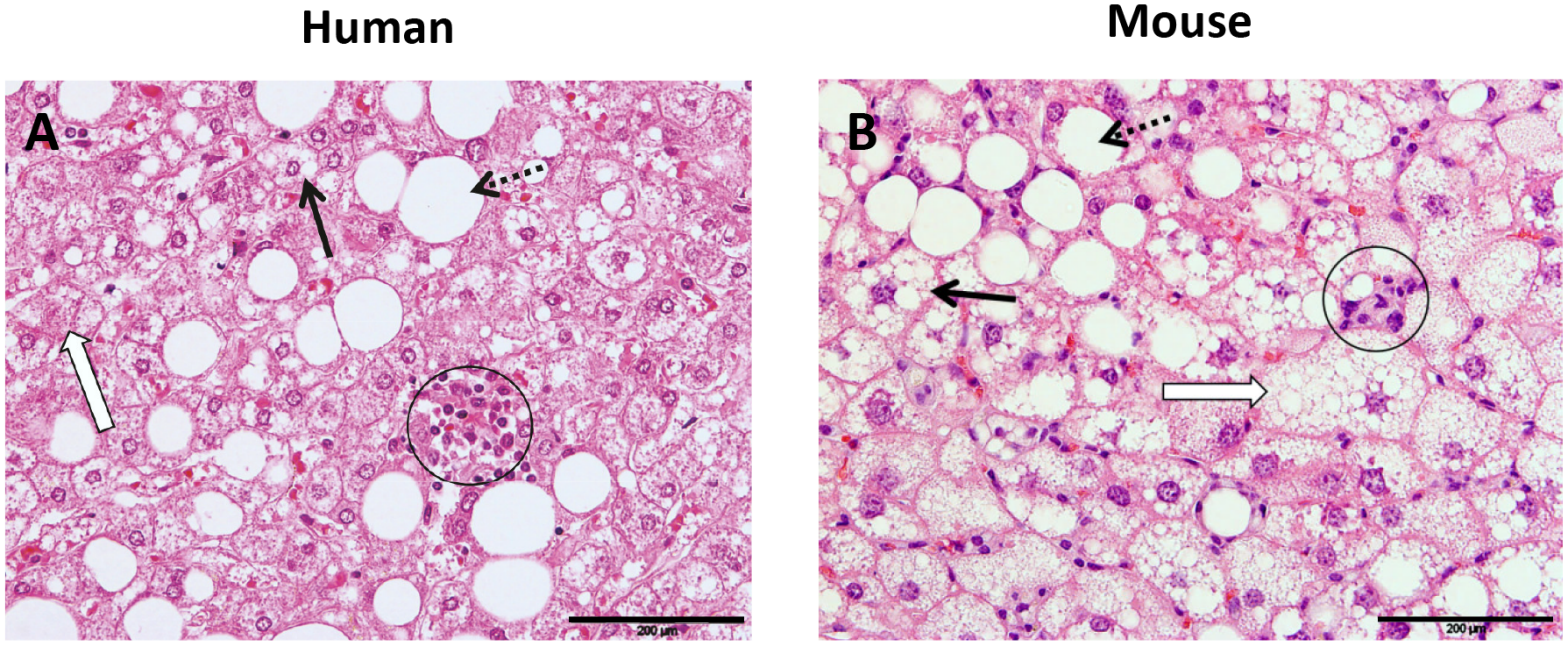
Hepatocellular steatosis, hypertrophy and inflammation. Liver cross-sections are from a NASH patient (A) or a E3L.CETP mouse fed a high fat diet supplemented with cholesterol to induce NASH (B). Macrovesicular steatosis (dotted line arrow): large lipid droplets are present in hepatocytes; microvesicular steatosis (bold arrow): small lipid droplets are present in hepatocytes. Hypertrophy (open arrow): the representative cell is much larger than the surrounding steatotic hepatocytes but has the same cytoplasmic characteristics. Clusters (aggregates) of inflammatory cells (within circles). All photomicrographs: Hematoxylin and eosin; magnification 200x.

Briefly, the two key features of NASH, steatosis and inflammation, were categorized as follows: steatosis was determined by analyzing hepatocellular vesicular steatosis, i.e. macrovesicular steatosis and microvesicular steatosis separately, and by hepatocellular hypertrophy as defined below (Fig. 2). Inflammation was scored by analyzing the amount of inflammatory cell aggregates (Fig. 2). The proposed rodent scoring system is shown in Table 4 and options for its use in diagnosis are shown in S1 Fig. The purpose of this scoring system is however not to derive a single score, but to score the individual features.

**Table 4:** Grading system for rodent NAFLD.

Histological feature		Score			
		0	1	2	3
Steatosis:					
	Macrovesicular steatosis	<5%	5–33%	33–66%	>66%
	Microvesicular steatosis	<5%	5–33%	33–66%	>66%
	Hypertrophy	<5%	5–33%	33–66%	>66%
Inflammation:					
	Number of inflammatory foci/field	<0.5	0.5–1.0	1.0–2.0	>2.0

Macrovesicular steatosis and microvesicular steatosis were both separately scored and the severity was graded, based on the percentage of the total area affected, into the following categories: 0 (<5%), 1 (5–33%), 2 (34–66%) and 3 (>66%). The difference between macrovesicular and microvesicular steatosis was defined by whether the vacuoles displaced the nucleus to the side (macrovesicular) or not (microvesicular). Similarly, the level of hepatocellular hypertrophy, defined as cellular enlargement more than 1.5 times the normal hepatocyte diameter, was scored, based on the percentage of the total area affected, into the following categories: 0 (<5%), 1 (5–33%), 2 (34–66%) and 3 (>66%). For hepatocellular hypertrophy the evaluation was merely based on abnormal enlargement of the cells, irrespective of rounding of the cells and/or changes in cytoplasm or the number of vacuoles, and is therefore not a substitute of ballooning. The unweight sum of the scores for steatosis (macrovesicular steatosis, microvesicular steatosis and hypertrophy) thus ranged from 0–9. Both steatosis and hypertrophy were evaluated at a 40 to 100× magnification and only the sheets of hepatocytes were taken into account (terminal hepatic venules and portal tracts etc were excluded).

Inflammation was evaluated by counting the number of inflammatory foci per field using a 100 x magnification (view size of 3.1 mm^2^). A focus was defined a cluster, not a row, of ≥5 inflammatory cells. Five different fields were counted and the average was subsequently scored into the following categories: normal (<0.5 foci), slight (0.5–1.0 foci), moderate (1.0–2.0 foci), severe (>2.0 foci).

Hepatic fibrosis was identified using Sirius Red stained slides at 40 x magnification and evaluated by scoring whether pathologic collagen staining was absent (only in vessels) or collagen staining observed within the liver slide, the latter further defined as mild, moderate or massive. In addition, the percentage of the total area affected was evaluated using using image analysis of surface area on Sirius red stained slides.

### Validation of histological NAFLD scoring system in mice

To validate the scoring system, 36 slides of mouse livers covering the whole spectrum of NAFLD, were blindly analyzed by a board-certified pathologist (A.L.M), a clinical pathologist (A.D.) and nine scientists with basic histological experience. For the validation, the observers estimated the percentage of macrovesicular steatosis, microvesicular steatosis and hypertrophy (relative scale) and the number of inflammatory foci per field (absolute scale), instead of using the different categories for steatosis and inflammation (ordinal measure). Additionally, quantification of the steatosis and inflammation was determined by one observer during two separate assessments that were separated by an interval longer than 3 months.

### Statistical analysis

The reproducibility of the scoring system was determined by calculating the Intraclass Correlation Coefficient (ICC) to determine inter-observer reliability among ten observers or the intra-observer reliability among two separate scoring assessments of one observer. Using two-way random model with absolute agreement and the 95% confidence interval (CI), ICCs were calculated for the agreement on NAFLD/NASH criteria of macrovesicular steatosis, microvesicular steatosis, hypertrophy and inflammation.

The ICC was interpreted as follows, according to the Munro classification system little or no correlation for values below 0.25, low correlation for values between 0.26–0.49, moderate correlation for values between 0.50–0.69 high correlation for values between 0.70–0.89 and very good correlation for values between 0.90–1.00 [13]. The Bland-Altman plot [14] was used to analyze inter- and intra-observer reproducibility to permit a better visualization of the correlation between individual measures. Results were considered significant at P-value<0.05 and statistical analyses were performed using SPSS version 20.0 (IBM, Somers, NY, USA).

## Results

### Development of histological NAFLD scoring system in mice

To define the overlapping NAFLD features in humans and mice, histological samples from several mouse NAFLD models were compared to human tissue biopsies of NAFLD patients. A summary of the main features of human NAFLD according to the Kleiner’s scoring system and their presence or absence in the mouse NAFLD models is shown in Table 1. Overall, the histological cross-sections of the mouse NAFLD looked similar to those of the human NAFLD patients (Fig. 1), but not all main features of human NAFLD were present in the mouse NAFLD models. The most important features of steatosis and inflammation, as well as fibrosis, were all found to be present in mouse NAFLD models as well. However, the features for hepatocellular injury, like apoptosis, pigmented macrophages and megamitochondria, were not found in any of the mouse NAFLD models. Ballooning, defined as enlarged and rounded hepatocytes with clear cytoplasm, was only occasionally found in the experimental NAFLD models, and was not prominently existing. Although enlarged hepatocytes were retrieved in all mouse NAFLD models, a distinct rounding of these cells concomitant with clear reticular cytoplasm was rarely found in all models.

Based upon the most important overlapping features, we selected the following main criteria for establishing a rodent NAFLD scoring system: 1) steatosis by determining the severity of macrovesicular steatosis, microvesicular steatosis and hypertrophy (as percentage of total area affected) and 2) inflammation by counting the number of inflammatory cell aggregates (as average number of five microscopic fields) (Fig. 2).

**Figure 3:**
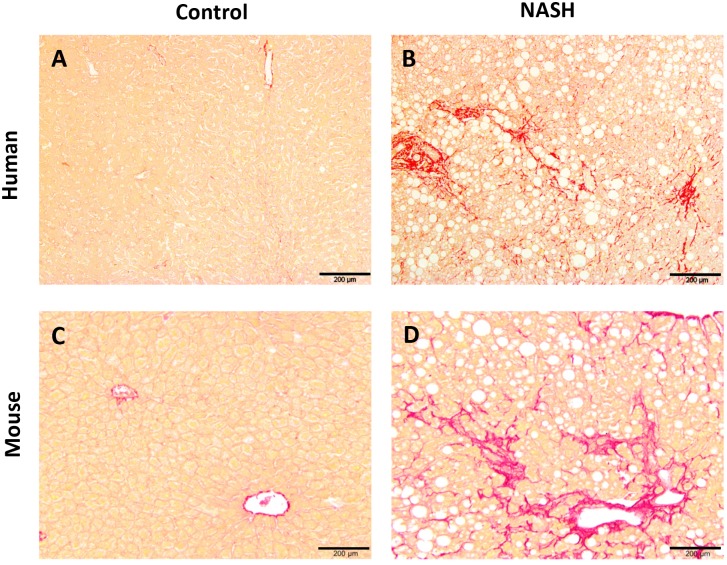
Liver fibrosis. Photomicrograph of cells with collagen staining (Sirius red). Liver histological cross-sections are from a healthy control subject (A) and a NASH patient (B) or a healthy control (C) and a LDLR^−/−^.Leiden mouse fed a high fat diet to induce NASH (D). All photomicrographs: Sirius red; magnification 100x.

In addition, the different staging of NAFLD was analyzed by identifying whether fibrosis was present and to which extent. In our present study, the most severe fibrosis was observed in the LDLR^−/−^.Leiden mice, followed by the E3L mice. In both models fibrosis was situated in both pericentral and perisinusoidal zone and sometimes bridging fibrosis was observed (Fig. 3).

### Validation of histological NAFLD scoring system in mice

**Figure 4:**
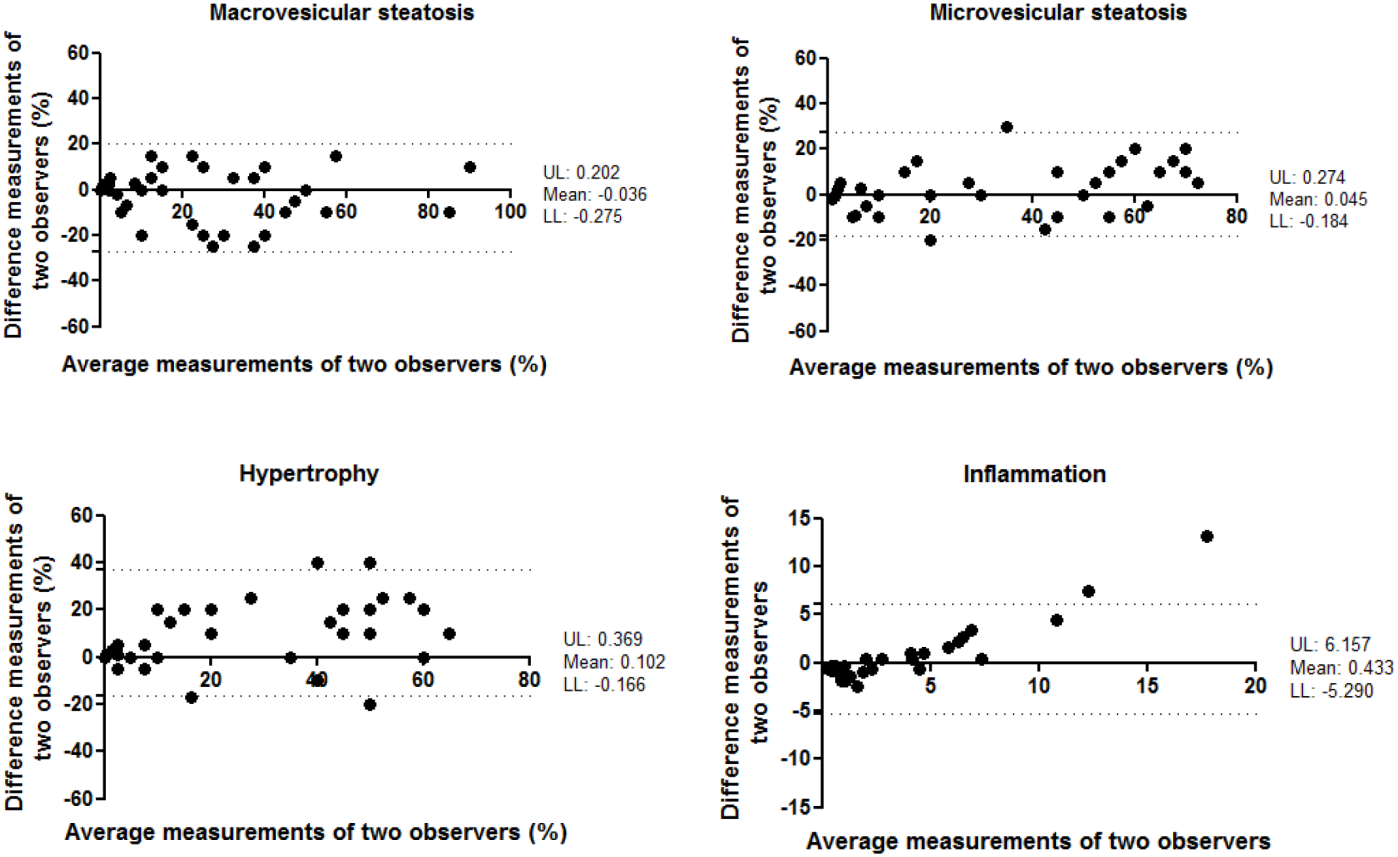
Bland-Altman plot for correlation analysis between two observers for measurement of macrovesicular steatosis, microvesicular steatosis, hypertrophy and inflammation. X-axis: average measurements of two observers. Y-axis: Difference between measurements of two observers. UL: Upper 95% limits of agreement. LL: Lower 95% limits of agreement.

A total of 36 mouse liver samples were used as validation set. This set of histological specimen covered the whole spectrum of NAFLD, from healthy unaffected samples to severe NASH livers with fibrosis (and included livers from various strains of mice). To evaluate the agreement between different observers, ten different observers analyzed the study set blindly. The level of inter-observer variation was subsequently evaluated using the Intraclass Correlation Coefficient (ICC). The ICC indicated a high correlation for the analysis of macrovesicular steatosis and microvesicular steatosis (ICC = 0.784 and 0.776, both p<0.001, respectively) and a moderate correlation for the analysis of hypertrophy and inflammation (ICC = 0.685 and 0.650, both p<0.001) between the different observers (Table 5). In conjunction with ICC, the Bland-Altman plots for two observers were also reported to visualize the correlation (Fig. 4). In addition, the agreement between two pathologists was examined. The ICC indicated a very good correlation for the analysis of microvesicular steatosis (ICC = 0.919, p<0.001), high for macrovesicular steatosis and hypertrophy (ICC = 0.814 and 0.784, both p<0.001, respectively) but low correlation for the analysis of inflammation (ICC = 0.471, p<0.01) between the two pathologists (S2 Table).

**Table 5:** Inter-observer reproducibility of NASH histological features according to the mouse NAFLD/NASH scoring system.

Histological feature	ICC	CI	P
Macrovesicular steatosis	0.784	0.681–0.869	<0.001
Microvesicular steatosis	0.776	0.679–0.860	<0.001
Hypertrophy	0.685	0.565–0.796	<0.001
Inflammation	0.650	0.517–0.779	<0.001

ICC: intraclass correlation coefficient (two-way random effects model, with absolute agreement); CI: 95% confidence interval; P: level of significance.

**Figure 5:**
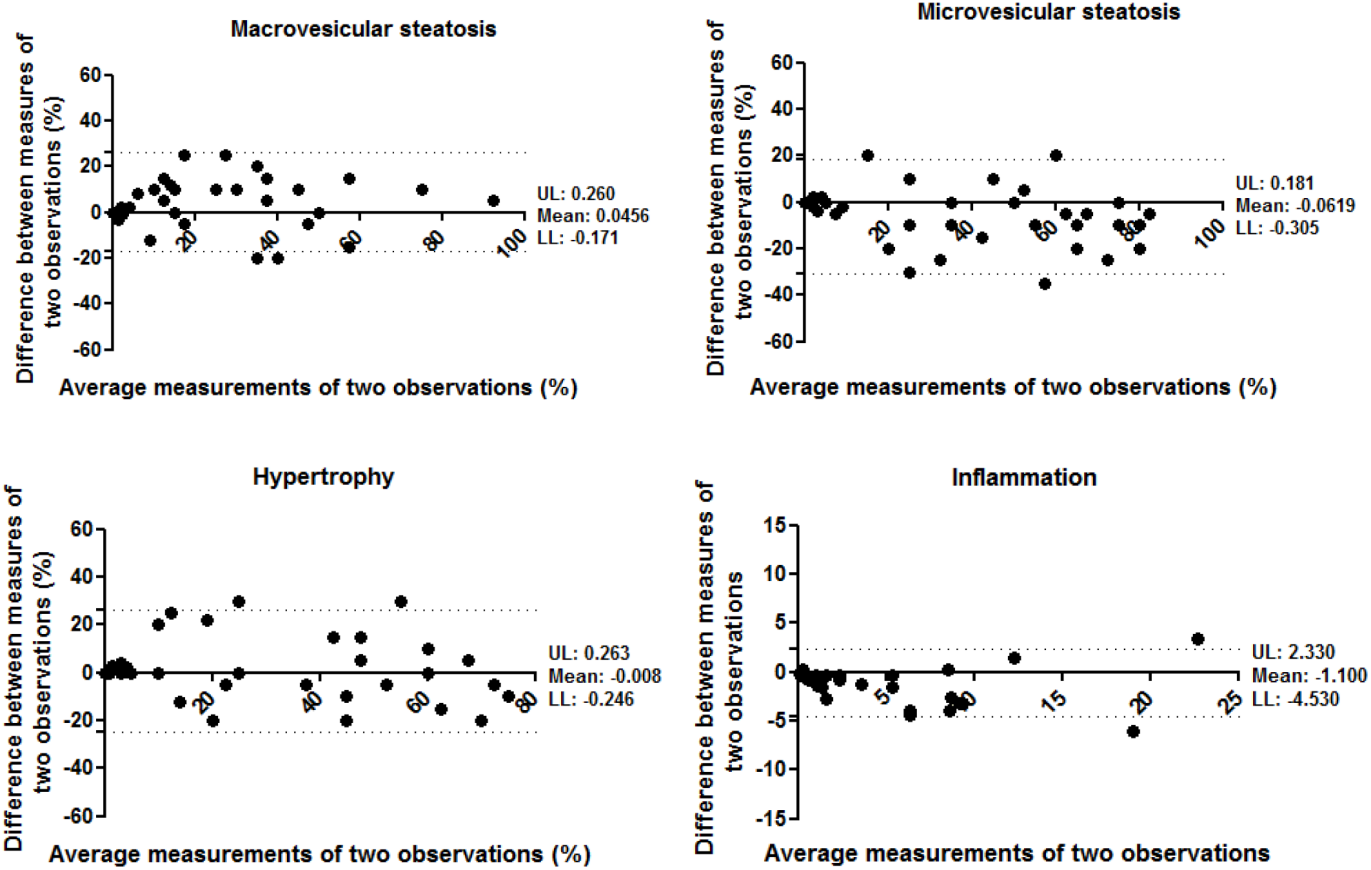
Bland-Altman plot for correlation analysis between two observations of one observer for measurement of macrovesicular steatosis, microvesicular steatosis, hypertrophy and inflammation. X-axis: average measurements of two observations. Y-axis: Difference between measurements of two observations. UL: Upper 95% limits of agreement. LL: Lower 95% limits of agreement.

The agreement between different observations of one observer was evaluated by analysis of the study set during two separate scoring assessments that were separated by an interval of more than 3 months. In the analysis of intra-observer reproducibility, the ICC indicated a high correlation for the analysis of macrovesicular steatosis, microvesicular steatosis and hypertrophy (ICC = 0.871, 0.871 and 0.896, all p<0.001, respectively) and a very high correlation for the analysis of inflammation (ICC = 0.931, p<0.001) between the different time points of analysis (Table 6). The Bland-Altman plots confirmed that a correlation was observed between the first and second scoring assessments (Fig. 5).

**Table 6:** Intra-observer reproducibility of NASH histological features according to the mouse NAFLD/NASH scoring system.

Histological feature	ICC	CI	P
Macrovesicular steatosis	0.871	0.729–0.937	<0.001
Microvesicular steatosis	0.871	0.740–0.935	<0.001
Hypertrophy	0.896	0.805–0.945	<0.001
Inflammation	0.931	0.802–0.971	<0.001

ICC: intraclass correlation coefficient (two-way random effects model, with absolute agreement); CI: 95% confidence interval; P: level of significance.

## Discussion

A uniform, well-defined grading system that accurately defines the severity of NAFLD is a prerequisite for diagnosis and treatment of NASH, but also for preclinical research and development of novel pharmaceutical treatments. This study systematically compared human NAFLD liver pathology with the experimental liver pathology of several NAFLD mouse models (with emphasis on diet-inducible NAFLD models with obese phenotype). We found that specific features of human NASH, like (macro- or microvesicular) steatosis, lobular and portal inflammation, microgranulomas, large lipogranulomas and fibrosis were present in the rodent models as well, but other features were absent (apoptosis, pigmented macrophages, megamitochondria, Mallory-Denk bodies and nuclear glycogenation) in the mouse NAFLD models or only occasionally retrieved (ballooning). In general, the NASH pathology of the mouse models was slightly less severe than the human NASH pathology, and many models mainly display early disease stages which makes them particularly suitable for the development of novel treatments that target the early processes of NASH or try to prevent fibrosis.

Our proposed scoring system (Table 4) is based on the features pertaining to mouse NAFLD and differs from the NAS scoring method for humans in that we omitted the assessment of ballooning and added the measurement of hepatocellular hypertrophy. Clinically, hepatic ballooning is a structural manifestation of microtubular disruption and hepatocyte injury [15]. Hepatic ballooning can be histopathologically identified as cells, which have rounded contours, a central and (small) pyknotic nucleus and two to three times the size of adjacent normal hepatocytes and that are characterized by a clear or wispy cytoplasm on H&E stained sections [15]–[17]. The underlying source of the swelling cannot be determined on H&E staining and therefore consensus on the underlying mechanism has remained elusive. The grading of ballooning into different scores has been difficult to perform and the definition has remained largely descriptive for the less advanced ballooning cells (grade 1) [18], [19]. As a consequence, ballooning is subject to significant inter-observer variation: there is often disagreement even among experts about the presence or absence of cellular ballooning in NASH [5], [6]. Although enlarged hepatocytes were retrieved in all mouse NAFLD models, distinct ballooning, was occasionally found in the models studied and under the conditions employed. The enlargement of the cells was primarily due to lipid accumulation, as confirmed via Oil-red-O staining (data not shown). Since ballooning can be detected by the loss of K8/18 in the cytoplasm, we performed a CK18 staining. In some hypertrophic cells a reduction, but not complete loss, in CK18 staining in the cytoplasm could be observed (S2 Fig.). We cannot rule out that either in other animal models or in the models at a more advanced stage of NASH, ballooning can occur more prominently. In order to define quantitatively measures we decided to omit ballooning from the proposed non-human, rodent NAFLD scoring system and to add hepatocellular hypertrophy. Importantly, hepatocellular hypertrophy is not a substitute of ballooning because, in contrast to ballooning, hypertrophy is not a sign of cellular injury, and merely refers to an abnormal enlargement of the cells without acknowledging the source of this enlargement.

In order to enhance the reproducibility of the scoring system, between different observers and between different observations of one observer, we kept our scoring system simple and did not take into account the zonal distribution pattern of the vesicular steatosis, hypertrophy and fibrosis nor with regards to inflammation, i.e. the type of inflammatory cells. Nevertheless, we do acknowledge the importance of this additional information and although not quantitatively embedded in our scoring system, we did evaluate the zonal patterns of the scored features and types of inflammatory cells in our models and found for C57BL6 on HFD, E3L, E3L.CETP, LDLR^−/−^ and KKA^y^ mice that the pericentral zone was most prominently affected thereby resembling the pathology in humans, whereas for C57BL6 on MCD diet predominantly macrovesicular steatosis around the periportal zone was observed. Furthermore, in all models with hepatic inflammation we found a mixture of inflammatory cells consisting of monocytes, Kupffer cells and polymorphic nuclear cells. Based on time course analyses in the models studied herein, MPO-positive immune cells (neutrophils) are typically found in the inflammatory cell aggregates which resembles the situation in humans [20], and indicates that tissue damage has occurred.

The NASH CRN scoring system is not including fibrosis in the activity score, but is using a separate grading system for fibrosis, based on perisinusoidal, periportal and bridging fibrosis or cirrhosis. Although fibrosis is not a requirement for the diagnosis of NASH, fibrosis is often present in NASH patients. Until recently, NASH was the only pattern within NAFLD that was recognized to be associated with the development of advanced fibrosis [21]. Fibrosis however can be unequally distributed throughout the liver and therefore sampling variability plays an important role in the diagnosis of clinical samples [22]. As expected, small biopsies (<1.6 cm) present higher variability for NAFLD fibrosis stage than larger biopsies [23]. Fortunately with (rodent) animal studies the whole liver can be analyzed, facilitating a proper pathological classification.

In our analysis, the rodent scoring system appears to be reproducible since concordance in the inter-observer and intra-observer scoring of macrovesicular steatosis and microvesicular steatosis were both high and moderate for hepatocellular hypertrophy, with better intra-observer scores than inter-observer scores for all features. In rodents the best measure of steatosis is biochemical quantification of hepatic triglycerides. True validation of the scoring system of steatosis would therefore be a correlation with biochemical triglyceride analysis. In several experiments, using different mouse strain and diet combinations, we correlated the scores of macrovesicular steatosis, microvesicular steatosis and hypertrophy with the biochemically measured hepatic triglycerides. In all experiments, we found a correlation, although interestingly in some mouse strain-diet combinations the correlation was better with microvesicular steatosis and in other mouse strain-diet combinations the correlation was better with macrovesicular steatosis. As an example the correlation of the samples used in the validation set (consisting of different mouse strains and different diets) for biochemical triglyceride measurement and microvesicular steatotis is shown in S3 Fig.). The inter-observer scoring of inflammation had the lowest concordance, while remarkably for the intra-observer scoring of inflammation the highest concordance was observed, suggesting that the scoring of this feature is the most subjective. These results corroborate the findings of several previous reproducibility studies that consistently report less agreement for scoring of inflammation [24]–[28]. The studies that also evaluated the inter-observer agreement all show better intra-observer scores than inter-observer scores [24], [25], [28].

One limitation of our study is that we based the rodent scoring system on the evaluation of five mouse strains and their comparison with the human features of steatosis and steatohepatitis. It remains to be seen whether the proposed rodent scoring system is applicable to other rodent models and whether the models used herein are representative for other models. However, since the basic features of NASH, namely steatosis and inflammation are displayed correctly in our scoring system, we expect the scoring system will be applicable for other animal models as well.

In conclusion, we established a simple, robust and generic NAFLD scoring system for rodents that is reliable and reproducible among observers. This study demonstrates its use in preclinical research using several rodent models which display different stages of the etiology of NAFLD.

## Supporting Information

S1 Fig **Diagnosis diagram for NAFLD.** The two key features of NASH, steatosis (0–9) and inflammation (0–3), were used in the proposed rodent scoring system.
(DOCX)

S2 Fig **K8/18 staining in liver slices.** Arrow: the loss of K8/18 in the cytoplasm in hypertrophic cells.
(DOCX)

S3 Fig **Correlation analysis of biochemical triglyceride measurement and microvesicular steatotis.** All samples used in the validation set (consisting of different mouse strains and different diets).
(DOCX)

S1 Table **Additional histological features of human samples per diagnosed category.**
(DOCX)

S2 Table **Inter-pathologist reproducibility of NASH histological features according to the mouse NAFLD/NASH scoring system.**
(DOCX)
