# Supplementary material for: Establishment of a General NAFLD Scoring System for Rodent Models and Comparison to Human Liver Pathology
Source: PLoS One. 2014 Dec 23;9(12):e115922. doi: 10.1371/journal.pone.0115922 (PMC4275274; doi:10.1371/journal.pone.0115922)
Supplement: S1 Fig — Diagnosis diagram for NAFLD. The two key features of NASH, steatosis (0–9) and inflammation (0–3), were used in the proposed rodent scoring system. (DOCX) [file pone.0115922.s001.docx]

**Figure S1:**

**Steatosis (0-9): Inflammation (0-3): Diagnosis:**

Macrovesicular steatosis (0, 1, 2, 3) (0, 1, 2, 3)

Microvesicular steatosis (0, 1, 2, 3)

Hypertrophy (0, 1, 2, 3)

0 0, 1, 2, 3 No NAFLD

1-9 0 NAFLD

1, 2, 3 NASH
