# Supplementary figures and images for: Establishment of a General NAFLD Scoring System for Rodent Models and Comparison to Human Liver Pathology
Source: PLoS One. 2014 Dec 23;9(12):e115922. doi: 10.1371/journal.pone.0115922 (PMC4275274; doi:10.1371/journal.pone.0115922)

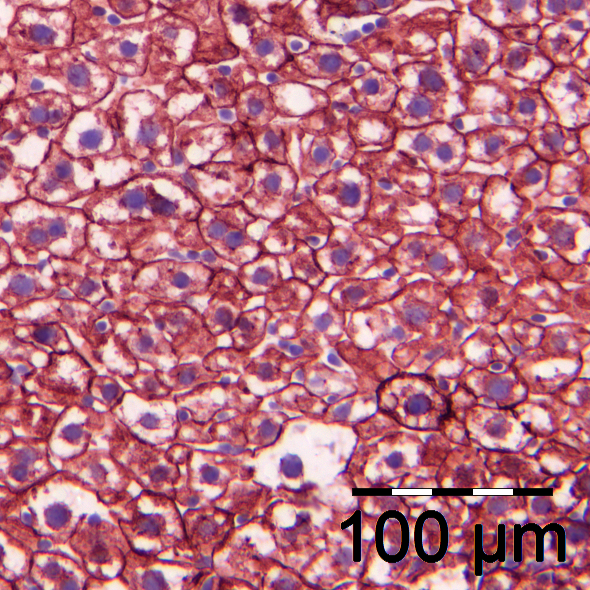
**Figure S2:**

Supplement: S2 Fig — K8/18 staining in liver slices. Arrow: the loss of K8/18 in the cytoplasm in hypertrophic cells. (DOCX) [file pone.0115922.s002.docx]
