# Supplementary material for: Establishment of a General NAFLD Scoring System for Rodent Models and Comparison to Human Liver Pathology
Source: PLoS One. 2014 Dec 23;9(12):e115922. doi: 10.1371/journal.pone.0115922 (PMC4275274; doi:10.1371/journal.pone.0115922)
Supplement: S1 Table — Additional histological features of human samples per diagnosed category. (DOCX) [file pone.0115922.s004.docx]

**Table S1:**

| **Histological feature** | **Score/code** | **Healthy (n=9)** | **NAFLD (n=11)** | **NASH (n=10)** |
| --- | --- | --- | --- | --- |
| Microgranulomas | 0 | 9 | 11 | 10 |
|  | 1 | 0 | 0 | 0 |
| Large lipogranulomas | 0 | 9 | 11 | 8 |
|  | 1 | 0 | 0 | 2 |
| Portal inflammation | 0 | 9 | 11 | 9 |
|  | 1 | 0 | 0 | 1 |
| Acidophil bodies | 0 | 9 | 11 | 10 |
|  | 1 | 0 | 0 | 0 |
| Pigmented macrophages | 0 | 9 | 11 | 7 |
|  | 1 | 0 | 0 | 3 |
| Megamitochondria | 0 | 9 | 11 | 8 |
|  | 1 | 0 | 0 | 2 |
| Mallory-Denk bodies | 0 | 9 | 11 | 10 |
|  | 1 | 0 | 0 | 0 |
| Glycogenated nuclei | 0 | 9 | 8 | 6 |
|  | 1 | 0 | 3 | 4 |

Number of subjects per score/code are shown for the indicated histological features.
