# Supplementary material for: Establishment of a General NAFLD Scoring System for Rodent Models and Comparison to Human Liver Pathology
Source: PLoS One. 2014 Dec 23;9(12):e115922. doi: 10.1371/journal.pone.0115922 (PMC4275274; doi:10.1371/journal.pone.0115922)
Supplement: S2 Table — Inter-pathologist reproducibility of NASH histological features according to the mouse NAFLD/NASH scoring system. (DOCX) [file pone.0115922.s005.docx]

**Table S2:**

| **Histological feature** | **ICC** | **CI** | **P** |
| --- | --- | --- | --- |
| Macrovesicular steatosis | 0.814 | 0.667–0.900 | <0.001 |
| Microvesicular steatosis | 0.919 | 0.848–0.957 | <0.001 |
| Hypertrophy | 0.784 | 0.619–0.883 | <0.001 |
| Inflammation | 0.471 | 0.145–0.704 | 0.003 |

ICC:_:_ intraclass correlation coefficient (two-way random effects model, with absolute agreement); CI: 95% confidence interval; P: level of significance.
